# Experiences from conducting systematic reviews of systematic reviews

**DOI:** 10.1186/s12874-026-02880-7

**Published:** 2026-06-10

**Authors:** Wilhelmus Johannes Andreas Grooten, Tony Bohman, Carina Bostrom, Åsa Dedering, Marie Halvorsen, Roman P. Kuster, Lena Nilsson‑Wikmar, Christina B. Olsson, Graciela Rovner, Elena Tseli, Eva Rasmussen‑Barr

**Affiliations:** 1 Division of Physiotherapy, Department of Neurobiology, Care Sciences and Society, Karolinska Institutet, Huddinge, Sweden; 2 Karolinska Hospital NKS - Allied Health Professionals, Theme: Women´s Health and Allied Health Professionals, Stockholm, Sweden; 3 School of Health and Welfare, Dalarna University, Falun, Sweden; 4 The Health and Medical Care Administration, Region Dalarna, Falun, Sweden; 5 Therapy Science Lab, Lake Lucerne Institute, Vitznau, Switzerland; 6 ACT Institutet Sweden, Research and Education, Vejbystrand, Sweden

**Keywords:** Physical therapy, Exercise therapy, Rehabilitation, Chronic musculoskeletal pain, Umbrella review, Evidence-based medicine

## Abstract

This commentary discusses recent experiences from conducting two systematic reviews (SRs) of SRs. We address the strengths and limitations of conducting an SR of SRs in the field of evidence-based medicine in musculoskeletal health. Despite methodological challenges, we believe that SRs of SRs add value to the research community.

## Introduction

Eleven researchers within our research group at the Karolinska Institutet [1] in the musculoskeletal research area have recently conducted two systematic reviews (SRs) of SRs of randomized controlled trials (RCT) regarding the effect of various exercise types on pain and disability levels in patients with chronic low back and neck pain [2, 3]. The rationale for applying this methodology was to summarize and establish the certainty of evidence on the efficacy of various exercise therapies commonly proposed for these conditions since different SRs show different results. With our experiences fresh in mind of conducting these two SRs of SRs, we find it important to discuss the strengths and limitations of conducting an SR of SRs. The value of SRs of SRs should be seen from the perspective of recent discussions [4, 5] that SRs are dependent on the data quality of the original studies included, which could significantly limit their validity. Considering this, determining the evidence through an SR of SRs might thus be even more challenging and raise concern about their added value in evidence-based medicine.

### Strengths with SRs of SRs

There are multiple reasons why SRs of SRs have a value. In 2019, the number of SRs was estimated to 80 SRs published daily [6] and is constantly rising with nearly 113 SR in 2022 [7], which makes it difficult for researchers, clinicians, and policymakers to keep updated. Thus, SRs of SRs enable easy access to a summary of the available literature. Moreover, by merging the results from several SRs, preferably by using meta-analyses, the certainty of evidence in a specific area becomes clearer [8]. For example, the results of one SR may show no significant difference between two interventions, while merging the results of several SRs might show a more granular picture. In addition, the findings from SRs of SRs may serve as an entrance point for clinicians and decision-makers in healthcare fields when developing new clinical guidelines. SRs of SRs can also show what has already been studied and thus may form a base for the recommendation that there is no need to further study certain interventions or, in contrast, that some interventions need additional investigations. To continue to study interventions that already have shown a high certainty of evidence might mean a waste of time, money, and, importantly, the unnecessary use of research participants, which is also an ethical dilemma for researchers. On the other hand, even if some interventions may be considered well studied, it still may be of interest to further investigate these with advanced methodologies, such as meta-syntheses or network analyses to determine the effect size of the interventions.

### Limitations with SRs of SRs

As with every methodology, SRs of SRs entail some limiting factors. Firstly, SRs of SRs can be seen as a sort of whispering game, and participant data can be altered during the processing of three publications (original study, SR, and SR of SRs), which might lead to so-called error propagation. The accuracy of the SR of SR findings is of course dependent on the accuracy and reporting quality of the original study data. When conducting an SR of SRs, it should not be necessary to go back to the original RCTs, but for the purpose of our SRs of SRs, we felt compelled to do so as some of the included SRs showed baffling results. It is important to be sure that the data are correct, and we encourage authors of SRs to increase their reporting accuracy using existing guidelines such as TIDier and CERT [9, 10]. Moreover, journal editors also have a responsibility that the data reported in their journals are correct and that the SRs follow the Prisma 2020 guidelines. As reviewers, we often have very short time frames to complete our review of a submitted manuscript. For SRs and especially SRs of SR, this means only very limited time to check the data and we suggest that the review time should be adjusted to the type of article submitted. Currently, artificial intelligence tools for data extraction are developing and perhaps reviewers could use them in the future to check the accuracy of the data in the tables. However, the available tools are not yet reliable and still require extensive human validation [11].

The value of the SR of SRs is evidently directly related to the quality of the included SRs. In our SR of SRs for low back pain, 36% of the included SRs were of very low-to-critically low quality, and in our SR of SRs for chronic neck pain, this was true for 52%. This, of course, relates to what appraisal tool is used [12], and the fact that the assessment of risk of bias is subjective. We discussed whether authors of SRs of SRs should exclude SRs with low quality to increase their own study quality. It is suggested that bringing forward SRs of SRs on only high-quality SRs is a passable way to provide valid evidence to clinicians [8, 13]. On the other hand, excluding SRs with lower quality might increase the risk that certain topics will be omitted because no high-quality studies and SRs have yet been published. This is likely to be the case for new, emergent topics.

The inter-rater reliability in study selection, data extraction and quality assessment when performing an SR of SRs can be a challenge. In our SRs of SRs, we worked in pairs of two and discussed disagreements in a consensus dialogue with a third researcher and/or with the entire research group. For our SRs of SRs, it was necessary to work with five pairs due to the amount of data, and this could have introduced some bias. Working in pairs is a strength and is recommended when conducting SRs [14], but there might still be some inter-assessment variabilities between the pairs. Therefore, consensus discussions in the full group were necessary, although very time-consuming. Moreover, we used the critical appraisal tool AMSTAR-2 [15] for the included SRs as recommended by Cochrane. One limitation with AMSTAR-2 is that comparison of the study quality between different SRs could be problematic since different reviewer pairs could appraise methodological flaws and weaknesses differently. Hence, there’s a need to conduct piloting assessments and we recommend having multiple consensus discussions in the full research group. In addition, neither AMSTAR-2, nor GRADE allow for a downgrade of the level of evidence due to obvious errors in the included SRs. This became evident in the process of extracting data from the included SRs when we, by chance, discovered several errors in the reviews compared to the original studies.

In our SRs of SRs, we detected large overlaps for some exercise types, e.g., motor control exercises and Pilates in low back pain, and Traditional Chinese Exercise and Yoga in neck pain. An overlap means that at least two published SRs include the same original article or multiple articles. Authors should be very careful in making conclusions on effectiveness, evidence or health policy when there are more SRs than original studies published on a specific topic [3]. We encourage future authors of SR of SRs to handle this issue appropriately using the methods proposed by Pieper et al., 2014, in which a “corrected covered area (CCA)” is calculated based on the frequency of repeated occurrences of the same paper in the SRs included in the SR of SRs [16]. Still, authors should be aware that one large RCT included in several SRs could influence the point estimates of the included MAs, affecting the overall results of the SR of SRs to a large extent.

A major challenge for SRs of SRs is to summarize populations, control groups, interventions, timelines, or outcomes described in different ways in SRs. This becomes particularly evident if the same original study is included in several SRs with different descriptions of the population and intervention. Therefore, following the PICOT structure and the current reporting guidelines, such as proposed by the EQUATOR Network is important to get the included data to be as homogenously described as possible [17]. If a systematic review is not conducted in compliance with these or the PRISMA 2020 guidelines, it is difficult to reach confidence in the results. We cannot interpret the findings correctly and trust significant findings of a systematic review if the quality of the review is substandard. This will affect clinical practice and healthcare policy to a large extent.

We thoroughly discussed the feasibility of summarizing the data using a meta-analysis and arrived at different conclusions. In our SR of SRs focusing on exercises for low back pain, we conducted solely a narrative synthesis due to the large heterogeneity among included SRs. However, in the SR of SRs addressing exercises for neck pain, we conducted both a narrative synthesis and a meta-analysis. The narrative analysis encompassed all SRs, whereas the meta-analysis was limited to SRs with sufficiently homogeneous data. We found that the findings from our meta-analysis strengthened the findings from the narrative analysis. We strongly believe that a narrative analysis has great value, but if feasible we advise future authors of SRs of SRs to consider performing a meta-analysis that could be used to strengthen the results and to follow the guidelines for analyzing heterogeneity [14, 18], even if it consists of a sub-sample of the included SRs. To establish the certainty of evidence on the efficacy of exercise interventions, our SRs of SRs were limited to SRs including predominantly RCTs. However, if one wants to investigate additional aspects of exercise interventions such as mechanisms of action explaining intervention effects or what psychological and psychosocial factors are of importance for a successful intervention outcome, other study types could provide equally valuable information. Reviews on these related topics should thus not be limited to RCTs.

## Conclusion

SRs of SRs can be an important tool to analyze the certainty of evidence in a particular research field. They provide a detailed overview of the available literature, might identify research gaps, and could serve as an entrance point when developing new clinical guidelines. However, the conduction of SRs of SRs is challenging. It is therefore important that authors consider data verification to limit error propagation (from the original study to SR to SR of SRs), to define how to deal with low-to-critically low-quality SRs, and to assess overlap (multiple SRs including the same original studies). We also recommend working in pairs but setting off or planning enough time for consensus discussions in the group of authors. Future review articles on this topic might also consider including a broader spectrum of study types to summarize evidence on potential mechanisms or psychological and psychosocial factors of importance for successful interventions.

## Data Availability

No datasets were generated or analysed during the current study.

## Abbreviations

AMSTAR: Assessing the Methodological Quality of Systematic Reviews
CCA: Corrected Covered Area
EQUATOR: Enhancing the QUAlity and Transparency Of health Research
SR: Systematic Review
PICOT: Population, Intervention, Control group, Outcome and Time
RCT: Randomized Controlled Trials

## References

[1] Grooten W. Musculoskeletal disorders; planetary health & biopsychosocial approaches – Nina Brodin's research group. 2026. Available from: https://ki.se/en/research/research-areas-centres-and-networks/research-groups/musculoskeletal-disorders-planetary-health-biopsychosocial-approaches-nina-brodins-research-group#tab-start.

[2] Grooten W, Bostrom C, Dedering A, Halvorsen M, Kuster R, Nilsson-Wikmar L, et al. Summarizing the effects of different exercise types in chronic low back pain - a systematic review of systematic reviews. BMC Musculoskelet Disord. 2022;23(1):801. 35996124 10.1186/s12891-022-05722-x PMC9394044

[3] Rasmussen-Barr E, Halvorsen M, Bohman T, Bostrom C, Dedering A, Kuster RP, et al. Summarizing the effects of different exercise types in chronic neck pain - a systematic review and meta-analysis of systematic reviews. BMC Musculoskelet Disord. 2023;24(1):806. 37828488 10.1186/s12891-023-06930-9 PMC10568903

[4] Jull G, Moore A. Systematic reviews in review. Musculoskelet Sci Pract. 2020;46:102135. 32217267 10.1016/j.msksp.2020.102135

[5] Horton R, Offline. The gravy train of systematic reviews. Lancet. 2019;394(10211):1790. 31741445 10.1016/S0140-6736(19)32766-7

[6] Hoffmann F, Allers K, Rombey T, Helbach J, Hoffmann A, Mathes T, et al. Nearly 80 systematic reviews were published each day: Observational study on trends in epidemiology and reporting over the years 2000–2019. J Clin Epidemiol. 2021;138:1–11. 34091022 10.1016/j.jclinepi.2021.05.022

[7] Andersen MZ, Zeinert P, Rosenberg J, Fonnes S. Comparative analysis of Cochrane and non-Cochrane reviews over three decades. Syst Rev. 2024;13(1):120. 38698429 10.1186/s13643-024-02531-2 PMC11064235

[8] Smith V, Devane D, Begley CM, Clarke M. Methodology in conducting a systematic review of systematic reviews of healthcare interventions. BMC Med Res Methodol. 2011;11(1):15. 21291558 10.1186/1471-2288-11-15 PMC3039637

[9] Hoffmann TC, Glasziou PP, Boutron I, Milne R, Perera R, Moher D, et al. Better reporting of interventions: template for intervention description and replication (TIDieR) checklist and guide. BMJ. 2014;348:g1687. 24609605 10.1136/bmj.g1687

[10] Slade SC, Finnegan S, Dionne CE, Underwood M, Buchbinder R. The Consensus on Exercise Reporting Template (CERT) applied to exercise interventions in musculoskeletal trials demonstrated good rater agreement and incomplete reporting. J Clin Epidemiol. 2018;103:120–30. 30055247 10.1016/j.jclinepi.2018.07.009

[11] Blaizot A, Veettil SK, Saidoung P, Moreno-Garcia CF, Wiratunga N, Aceves-Martins M, et al. Using artificial intelligence methods for systematic review in health sciences: A systematic review. Res Synth Methods. 2022;13(3):353–62. 35174972 10.1002/jrsm.1553

[12] Perry R, Whitmarsh A, Leach V, Davies P. A comparison of two assessment tools used in overviews of systematic reviews: ROBIS versus AMSTAR-2. Syst Rev. 2021;10(1):273. 34696810 10.1186/s13643-021-01819-x PMC8543959

[13] Roberts I, Ker K. How systematic reviews cause research waste. Lancet. 2015;386(10003):1536. 26530621 10.1016/S0140-6736(15)00489-4

[14] Higgins JPT, Thomas J, Chandler J, Cumpston M, Li T, Page MJ et al. Cochrane Handbook for Systematic Reviews of Interventions Version 6.1 (updated September 2020) 2020. Available from: https://www.training.cochrane.org/handbook.

[15] Shea BJ, Reeves BC, Wells G, Thuku M, Hamel C, Moran J, et al. AMSTAR 2: a critical appraisal tool for systematic reviews that include randomised or non-randomised studies of healthcare interventions, or both. BMJ. 2017;358:j4008. 28935701 10.1136/bmj.j4008 PMC5833365

[16] Pieper D, Antoine SL, Mathes T, Neugebauer EA, Eikermann M. Systematic review finds overlapping reviews were not mentioned in every other overview. J Clin Epidemiol. 2014;67(4):368–75. 24581293 10.1016/j.jclinepi.2013.11.007

[17] The EQUATORN. [2024-05-14]. Available from: https://www.equator-network.org/reporting-guidelines/tidier/.

[18] Schünemann H, Brożek J, Guyatt G, Oxman A, editors. GRADE handbook for grading quality of evidence and strength of recommendations. The GRADE Working Group, 2013. 2013. Available from guidelinedevelopment.org/handbook.

